# Mental health literacy and academic performance (MHLAP) in high school students: a randomized clinical trial protocol

**DOI:** 10.1186/s13063-024-08270-y

**Published:** 2024-06-27

**Authors:** Cezar Giosan, Alexandru Pană, Ana Cosmoiu, Ana-Maria Chira, Ana-Maria Toma, Claudiu-Cristian Papasteri, Cătălin Nedelcea, Cătălina Popoviciu

**Affiliations:** University of Bucharest, Bucharest, Romania

**Keywords:** Mental health literacy, Academic performance, Mental health, Randomized clinical trial

## Abstract

**Background:**

Mental health literacy is a promising avenue of intervention for addressing the development of psychopathology, as well as its associated consequences, such as a decrease in academic performance. The current study aims to test the effectiveness of such an intervention in high school students, focusing on two main formats of delivery: (1) automated and (2) blended.

**Methods:**

To achieve this aim, a randomized clinical trial with direct comparisons at three time points between three conditions (automated, blended, and waitlist) was designed. Power analyses yielded a necessary sample size of 264 high school students. The participants will be selected from Romanian high schools.

**Discussion:**

The current study aims to contribute to the mental health literacy literature by testing the effectiveness of an educational intervention concerning mental health in terms of its benefits for reducing psychopathology and increasing academic performance. The success of such an intervention bears important implications for addressing mental health in the educational system.

**Trial registration:**

Clinicaltrials.gov Identifier: NCT06217744, version 1, 22 January 2024.

**Supplementary Information:**

The online version contains supplementary material available at 10.1186/s13063-024-08270-y.

## Mental health literacy and academic performance

### Background

Mental disorders represent one of the most important risk factors associated with disability, accounting for more than a quarter of the responsible causes for disability in people between the ages of 15 and 44 [1]. The worldwide prevalence of any mental disorder in children and adolescents is estimated at 13.4% [2]. Mental disorders are associated with school absenteeism [3, 4], academic-related stress and poorer academic performance, learning capacity, and physical and mental health outcomes [5].

Even though there are effective mental health interventions for the youth, such as psychotherapy [6], canine-assisted psychotherapy [7], or psychiatric medication [8], to name a few, there are accessibility barriers, ranging from geographical challenges to awareness of such problems in these populations. Some of the main reasons why adolescents avoid seeking mental health help include perceived stigma and shame associated with help-seeking and insufficient mental health-related knowledge [9]. At the same time, a history of pleasant experiences with mental health services and mental health literacy constitutes some of the most important facilitators for seeking professional help [10].

This clinical trial aims to address the aforementioned issues by testing the effectiveness of an intervention meant to increase mental health literacy in adolescents through a curriculum available at school level.

### Framework and rationale of the present study

Mental health literacy refers to having knowledge of how to recognize, deal with and prevent mental disorders [11]. Mental health literacy in adolescents can help develop support from parents, friends, or other members in a community, by doing so greatly complementing mental health services [12].

Unsurprisingly, poorer mental health literacy is associated with increased psychopathology, such as depression [13, 14], anxiety, stress, and other mental health outcomes [14]. There have been efforts to develop mental health literacy interventions, such as The Youth Education and Support (YES) Program, which is geared to grades 5–8 [15]. Although there is a wide range of mental health literacy interventions [16–18], most of them are delivered in a classroom format [19, 20], even though delivery through the Internet could be a viable option [16].

Most of the research in this field has been conducted in Australia, and it is unclear to what extent those findings are generalizable to different cultures and economically diverse countries [16]. The current MHL intervention is informed by "The Mental Health & High School Curriculum Guide" [21], an evidence-based mental health curriculum initially developed in Canada. Our intervention follows the structure of the aforementioned curriculum (details about the content of the intervention can be found in the “Methods” section of this paper). However, it is not a direct translation. Instead, it incorporates cultural adaptations to suit the Romanian context. Adjustments were made to address specific cultural factors in Romania. This includes adapting the language to reflect local stigmatizing words or expressions, addressing prevalent myths about mental disorders, and incorporating examples and role models relevant to Romanian teenagers. The adaptation process involved input from educational and mental health experts, school focus groups, and educational advocacy groups. The development of the intervention took 6 months. This period included extensive literature reviews, thorough documentation, and group discussions among professionals to ensure comprehensive coverage of key mental health issues relevant to teenagers (e.g., disorders usually starting in adolescence, such as eating disorders, or that might be of particular relevance in academic contexts, such as ADHD), appropriate and engaging language, and relevant examples tailored to their age and interests.

### Objectives

The goal of this study is to test the effectiveness of an intervention designed to promote mental health literacy in adolescents, in the format of a randomized clinical trial.

We aim to (1) determine whether this intervention has an impact on mental health, (2) observe if this intervention has an impact on fostering academic performance, particularly in disadvantaged students (i.e., academic resilience), and (3) examine what type of intervention delivery is more effective: automated versus blended format.

## Methods/design

### Trial design

This study is an exploratory randomized clinical trial involving comparisons at three time points (i.e., pre-intervention, post-intervention, 1 month follow-up) between three conditions: (a) automated intervention (consisting of an online, automated delivery of the intervention), (b) blended intervention (online automated delivery of the intervention plus regular contact with facilitators), (c) waitlist, with an allocation ratio of 1:1:1. The contrast between the automated and blended interventions is meant to highlight whether contact with facilitators while receiving psychoeducational materials might have an additive effect.

The design of this study complies with the Consolidated Standards of Reporting Trials (CONSORT) guidelines [22] and follows the Standard Protocol Items: Recommendations for Interventional Trials (SPIRIT) Statement 2013 [23] (see also Additional file 1: SPIRIT checklist and schedule of enrollment, interventions, and assessments).

The current trial is part of a larger project that aims to assess adolescent mental health in Romanian high schools.

### General description of the tested application

SCHOLARS is a web-based mental health literacy intervention containing six modules as follows: Module 1: The Stigma of Mental Illness; Module 2: Understanding Mental Health and Mental Illness; Module 3: Information on Specific Mental Illnesses; Module 4: Experiences of Mental Illness; Module 5: Seeking Help and Finding Support; Module 6: The Importance of Well-being. These modules will have both text and videos through which the users will progress. Both the automated and blended interventions are designed to last for three months, with two modules being delivered per month. Participants are encouraged to go through the materials at their own pace and have the option to revisit previous modules as needed. Participants can access the intervention from any location.

The intervention can be accessed through a LimeSurvey link and is compatible with various devices such as computers, tablets, and mobile phones. To ensure that the intervention will be properly delivered through this medium, we initially conducted a brief pilot test on a convenience sample consisting of university students. No issues were encountered during this phase.

Detailed information about the intervention is available on the project's website, which is linked on the initial page of the intervention. This resource allows participants to learn more about the study team responsible for the development of the intervention as well as the associated written and visual materials. Additionally, participants can ask any specific questions by emailing the research team; the email address is provided in the consent form.

### Study setting

The data will be collected from several high schools in Romania. They will be both state operated and privately owned. High schools will be enrolled based on their availability and willingness to participate in the study.

### Inclusion and exclusion criteria

The current trial will include Romanian high school students, specifically those in grades 9 through 12, aged 14 to 18 years old. Participation in the trial is open to all students within this age and grade range who have access to the internet, regardless of their Internet source. It does not matter where the Internet access comes from, be it from a home connection, school-provided resources, public libraries, or other means. The primary requirement is that these students can reliably access the Internet to participate in the study, ensuring that all eligible participants can engage. Of note is that, in 2021, Romania was ranked on the 5th place in the world in terms of Internet speed and, according to Eurostat, boasts one of the highest Internet penetration rates in Europe [24]; therefore, access to the Internet of our target population is not problematic.

### Study conditions

#### Experimental—automated intervention

This intervention will last for 3 months, with two psychoeducational modules delivered per month. The participants will receive a total of 6 modules, which will include both text and video-delivered information, as well as quizzes and practical activities.

#### Experimental—blended intervention

This intervention will last for 3 months, with two psychoeducational modules delivered per month. The participants will receive a total of 6 modules, which will include the same content as in the educational intervention. In addition, the participants will also have the possibility to engage with a facilitator on a regular basis, either via online live sessions (e.g., via Skype) or on a forum where they can ask questions and receive replies from the team.

### Sample size

R version 4.2.2 for Windows (R Foundation for Statistical Computing, Vienna, Austria) was used for the sample size calculation in a linear mixed-effect model (sjstats package). The R statistical computing environment will also be used for future model fitting and evaluation.

We used appropriate methods for two-level-designs [25] to determine sample size requirements for a cRCT with 3 experimental groups, 4 classrooms per experimental condition, 22 participants per cluster. For our linear mixed-effects model, a sample size of approximately 88 participants per experimental condition for a total sample size of 264 was sufficient to achieve 80% power to detect an effect size of Cohen’s *d* = 0.4 at an alpha level of 0.05. A medium effect size was assumed based on previous work on digital mental health literacy interventions [26].

### Recruitment

Recruitment will be made from various high schools in Romania.

### Assignment to study group

**Fig. 1:**
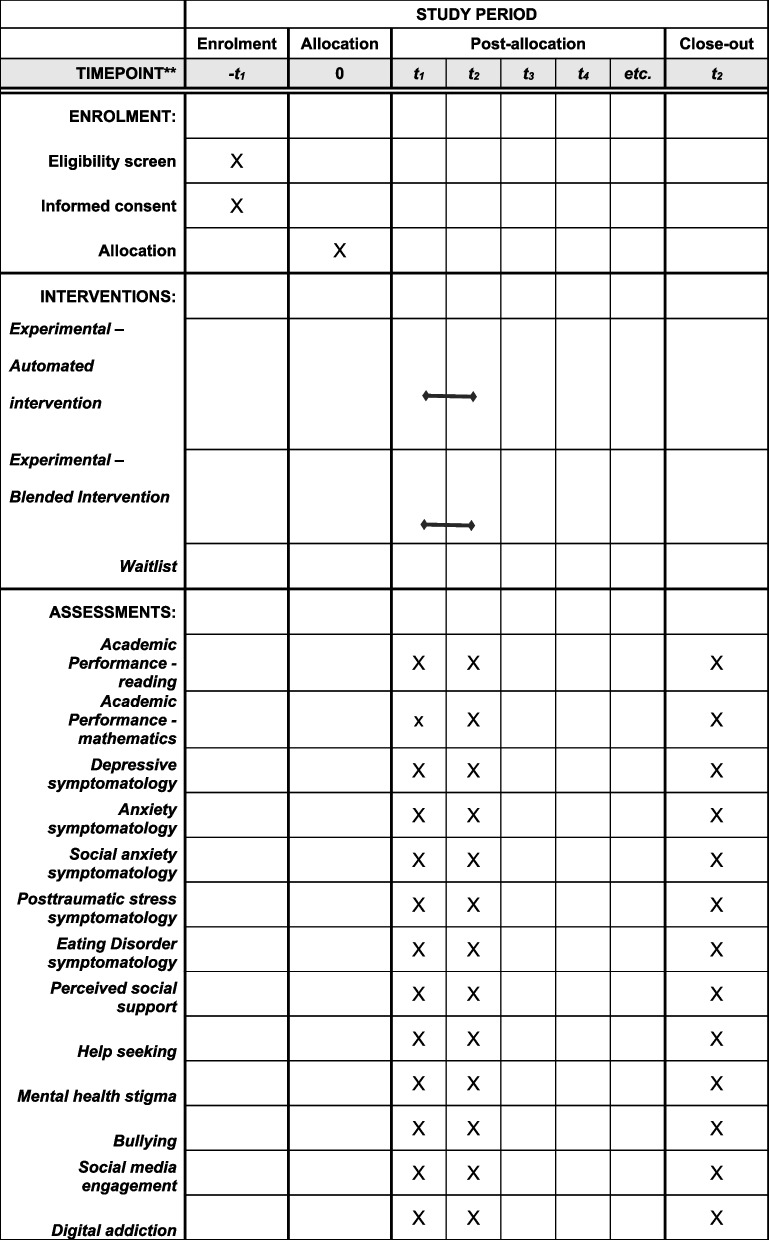
SPIRIT figure highlighting the proposed timeline of the intervention and assessments

The participants will be randomly assigned to one of the three trial arms (see Fig. 1). Randomization will be performed by a research assistant using a simple (unrestricted) randomization sequence that assigns one of three unique numbers (1, 2, or 3) per classroom enrolled in the intervention, signifying the experimental condition. No stratification will be employed. To conceal the allocation mechanism, the same research assistant will use sequentially numbered, opaque, sealed envelopes and will subsequently monitor the assessments and allow access to the application for the participants in the waitlist control group after 6 weeks. Two different research assistants will be responsible with enrolling the participants in the intervention and assigning them to the intervention by maintaining direct contact with student representatives from each classroom that is enrolled in the intervention. Neither the participants nor the psychologists involved in disseminating the intervention to the adolescents will be blinded, as blinding is not feasible given the context of the current trial. The principal investigator and the statisticians curating the dataset and running the data analysis will remain blinded to the study condition until the completion of the study.

### Data collection, management, and analysis

#### Data collection methods

The primary outcome measures are related to academic performance, well-being, and symptomatology, which will be collected at three time points: pre-intervention, post-intervention, and 1 month follow-up. Academic performance (math and reading comprehension) will be assessed with a sample of items taken from TIMSS (mathematics) and from PIRLS (reading comprehension) [27].

#### Measures administered

All the measures included in the current study are self-report questionnaires that have been selected after a careful review of the scientific literature for their appropriateness for being used with adolescents and young people as well as their validity and reliability.

##### Screening

The screening scale contains 26 items and target symptoms not covered by other scales in the instrument [28]. The first 22 items concern how much or how often participants have been affected by a series of situations in the two weeks before completion. The last four items cover substance use and suicidal thoughts, and participants are instructed to answer “Yes” or “No.”

##### Attention deficit hyperactivity disorder (ADHD)

To assess ADHD symptomology, the World Health Organization Adult ADHD Self-Report Scale (ASRS) [29] is used. It consists of six items, and, while it has been developed for adults, it is also suitable for use in adolescents. Participants are asked to indicate how often they have experienced certain situations for the first eight items and to indicate the approximate age when problems with organization, concentration, or impulsivity first occurred, if applicable, at the last item.

##### Depressive symptomatology

The PHQ-9-A Depression Scale (PHQ-A) [30] is the adolescent-adapted version of the Patient Health Questionnaire and contains ten items. Participants are instructed to rate on a scale of 0 to 4 how often they felt affected by certain problems and the extent to which these situations (if applicable) affected the respondent professionally, personally, or socially.

##### Anxiety

The anxiety rating scale [31] consists of eight items. Participants are asked to indicate on a scale from 0 to 4 how often they felt affected by certain problems and to rate the extent to which these situations (if applicable) affected the respondent professionally, personally, or socially.

##### Social anxiety

The Severity Measure for Social Anxiety Disorder (SMSAD 11–17) is a ten-item instrument for children and adolescents [32]. On a Likert-type scale from 0 to 5, participants are asked to indicate how often they experienced various emotions, thoughts, and behaviors in social situations.

##### Adverse childhood experiences

The Adverse Childhood Experiences Rating Scale employed in this study [33] is composed of two sections, totaling 19 statements. Participants are instructed to indicate the number of statements that accurately describe their situation growing up.

##### Posttraumatic stress

The Post Traumatic Symptom scale [34] consists of 37 items. The first part of the scale presents a series of 17 stressful situations and participants are asked to indicate whether they have been exposed to one or more of these events in their lifetime. The last 20 items refer to the individual's reaction after experiencing a stressful situation.

##### Bullying

Bullying will be assessed with the six items used in the World Mental Health Initiative [35]. The items refer to times when someone is intentionally hurt or scared by a peer in the school setting, on the Internet, and in close relationships.

##### Eating disorders

The NEDA Eating Disorder Screening Tool [36] is composed of 14 items. The first 11 items address different aspects of eating behavior and/or self-image. These contain multiple response options, with participants being guided to choose the one that suits them. The last three items refer to information about height, lowest weight in the last year, and current weight.

##### Social support

The Perceived Social Support Questionnaire [37] contains six items, measured on a five-point Likert-type scale, where 1 = Does not fit me at all and 5 = Fits me extremely well.

##### Seeking help

The Attitudes Toward Seeking Professional Psychological Help – Short Form [38] instrument assesses attitudes towards seeking specialized psychological help. It consists of ten items and participants are asked to express the extent to which they agree with the statements.

##### Mental health stigma

The Peer Mental Health Stigmatization Scale [39] assesses stigmatizing attitudes of people experiencing mental health problems. It is composed of 24 items and participants are asked to express the extent to which they agree with the items.

##### Social media engagement

Social Media engagement will be measured with the Social Media Engagement Questionnaire (SMEQ) [40]. The questionnaire contains 5 items measured on an 8-point Likert scale (0 = not one day; 7 = every day). Higher scores represent higher engagement with social media.

##### Digital addiction

Digital Addiction will be measured with the Digital Addiction Scale for Teenagers (DAST) [41]. This is a 10-item instrument measured on a 7-point Likert scale ranging from never to very often.

#### Statistical methods

Our study will consist of a multisite cluster randomized trial in which the unit of measurement is at student-level, while randomization takes place at the unit of assignment, at classroom-level. Consequently, it is a low bias cluster-level RCT with potential joiners (i.e., individuals not in intervention clusters at random assignment but who enter later) being excluded and scant possibility of “contamination” or “treatment spillover” to the control classrooms. Analyses will employ linear mixed model with individual-level repeated measures nested within classrooms, nested within schools. Adhering to WWC group design standards [42], the model will include indicators for each intervention period and interaction terms for assignment to the intervention group and the period associated with the intervention. We will also report on robust standard errors from our fitted models to adequately account for dependence within clusters.

Nevertheless, youth research, and especially youth mental health research, has unique challenges concerning data gathering. First, although we adhere to a strategic research design to reduce missing data, patterns of attrition relating to individual and cluster-level factors will be investigated, including links to social and health variables. Second, challenges in gathering parental consent after classroom-level assignment to experimental condition would yield higher number of leavers, which constitutes individual-level attrition [42]. If data is missing at random, our analytic choice will yield unbiased estimates of treatment effects [43]. If data is missing not at random, weighting and multiple imputation would be used. In line with WWC standards [42] for cluster-level assignment RCTs, we will employ regression imputation, maximum likelihood, or nonresponse weights to address missing values while providing evidence that the approach appropriately adjusts the standard errors for clustering.

##### Auditing

The Project Management team will meet monthly to discuss trial implementation or, upon necessity, if any potential difficulties arise. Additionally, the ethics committee is updated 6 months after the start of the trial or in the case that any changes to the trial protocol appear. The monitoring of unintended affects and inadvertent harm (e.g., unexpected, serious increases in symptomatology) will be undergone by team members who are also clinical psychologists and who will make referrals for appropriate interventions (i.e., clinical evaluation or psychotherapy), at which point the intervention will be discontinued immediately. Additionally, any such events will be appropriately and expeditiously reported to the ethics committee at the University of Bucharest.

The team responsible for recruitment and administrative tasks in the trial meets bi-weekly. The group consists of the principal investigator, project leader, and staff responsible for recruitment activities. The project leader, along with a research assistant, will monitor the trial, ensuring the eligibility of potential recruits, checking for the presence of informed consent, documentation of inclusion/exclusion criteria, and randomization for each participant. The project leader and research assistants will maintain constant communication with high schools and other relevant stakeholders.

### Ethics and dissemination

This study is approved by the ethics commission at the University of Bucharest. Since the recipients of the intervention are minors, careful consideration has been given to obtaining informed consent from parents or legal guardians, while also properly informing the students about the contents of the intervention that they are enrolled in. Participant data will be confidential and stored on secured servers. Additionally, since we also collect mental health data, special attention will be given to any increase in symptomatology or any participants presenting harm to themselves. If such situations arise, they will be handled by the clinical psychologists in the research team. Any subsequent changes to the protocol will be updated on ClinicalTrials.gov.

### Dissemination policy

We plan to publish the trial results in a peer-reviewed journal. Preliminary results will also be presented at international conferences, when appropriate. Additional plans are being made for the publication of the intervention manual. A summary of the results will also be made available for relevant stakeholders (i.e., high schools). Anonymized datasets will be made available upon reasonable request from the corresponding author.

## Discussion

The current trial aims to test the effectiveness of a mental health literacy intervention for high school students, aimed at improving academic performance, as well as increasing well-being and decreasing symptomatology. The intervention will be delivered via two different formats: (1) an educational one, which will be fully automated, and (2) a blended one which will additionally allow contact with facilitators, and it will be contrasted against a waitlist condition. Testing mental health literacy interventions is paramount, as not much is known about their effectiveness on outcomes such as academic performance and symptomatology. Furthermore, since the goal of mental health literacy interventions is to fill in the gaps that other mental health interventions cannot, in terms of their reach and cost-effectiveness, it also becomes highly relevant to test various modes of implementing such interventions, including fully automated ones. Additionally, testing the effectiveness of such interventions in varied cultural and socioeconomic contexts is timely, as this has been previously recognized as a limitation of the literature [19].

This endeavor, however, is not without its caveats. Firstly, contrasting active interventions against a waitlist control group can artificially inflate effect sizes. However, this approach is more ecological and cost-effective. If results will favor the SCHOLARS intervention, future studies should contrast it against active control conditions or psychological placebos. Secondly, there may be limited adherence to the intervention by the participants (i.e., dropouts). To this end, we aim to monitor and maintain close and constant contact with the enrolled schools, as to minimize drop-out and the risk of non-adherence by participants.

To conclude, the current study has the potential of advancing the field of mental health literacy interventions for adolescents. The success of such interventions might represent an important avenue for preventively addressing the development of mental health concerns and their associated consequences such as decreased academic performance.

### Trial status

Trial registration: https://clinicaltrials.gov/study/NCT06217744, Version 1, 22 January 2024. Recruitment for the trial has begun. Recruitment completion estimated date: November 2024.

### Supplementary Information

Supplementary Material 1.

## Data Availability

The datasets analyzed during the current study and statistical code used to analyze the data will be made available from the corresponding author upon reasonable request.
